# University of Buffalo Commencement

**Published:** 1904-06

**Authors:** 


**A Monthly Review of Medicine and Surgery.**

*EDITOR:*

WILLIAM WARREN POTTER, M. D.

All communications, whether of a literary or business nature, books for review and exchanges should be addressed to the editor: 284 FRANKLIN STREET, BUFFALO, N. Y.

VOL. XLIII.—LIX. JUNE, 1904. NO. 11

### University of Buffalo Commencement.

THE fifty-eighth annual commencement of the medical department was held May 3, 1904, at the Teck Theater, at 11 o'clock a. m. At the same time and place and conjointly therewith the departments of pharmacy and dentistry held their graduating exercises.

ALUMNI ASSOCIATION.

In the morning at 9.30 o'clock the alumni gathered at the University building, where several class reunions were held and the usual greetings interchanged. Many old friends met and polished the links of a somewhat rusty chain of earlier acquaintanceship, and many new friendships were formed. The several committees were busy with their duties, and the registration was large. At 11 o'clock adjournment was had and the members of the association proceeded in a body to the Teck Theater to attend the commencement exercise. Elsewhere we publish the proceedings of both morning and afternoon sessions in detail as reported by the secretary.

MEETING OF THE COUNCIL.

Early in the day the annual meeting of the council of the university was held. The vice-chancellor, Hon. George Gorham, presided and the recommendation of the several faculties relating to the candidates for the degrees in medicine, pharmacy, and dentistry was confirmed. Five vacancies had occurred in its own ranks since the last annual meeting, caused by resignation and death—namely, General John C. Graves, resigned, and James O. Putnam, George S. Hazzard, Worthington C. Miner, and William S. Bissell (chancellor), deceased. Stephen M. Clement,

L. L. Babcock, John B. Olmstead, J. L. O'Brian and R. R. Hefford were chosen to fill these vacancies.

The council also elected the following-named physicians to fill vacancies on the board of curators in the medical department: H. J. Nichols, Warren, Pa.; H. J. Knickerbocker, Geneva; Joseph Fowler, Benjamin G. Long and Franklin W. Barrows, Buffalo.

EXERCISES AT THE TECK.

A large audience assembled promptly at the appointed hour to greet the graduates and witness the ceremonies attendant upon the conferring of degrees. The several classes, numbering about 160, occupied seats immediately behind the orchestra, and the swells of music continued during the seating process. The faculties, lead by the Honorable George Gorham, vice-chancellor, then appeared upon the stage and took their appointed places.

DEPARTMENT OF MEDICINE.

The ceremonies began with prayer by the Rev. George B. Richards, rector of the Church of the Ascension, and after a musical selection the class in medicine, fifty-five in number, was presented to the chancellor by Professor John Parmenter, M.D., secretary of the faculty; then the dean, Professor M. D. Mann, M.D., administered the Hippocratic oath, after which the degree of doctor in medicine was conferred by the vice-chancellor, the Hon. Mr. Gorham, upon each of the following-named successful candidates:

Jabez Eldredge Armstrong, A. B.; Parker G. Borden, Fred Spencer Brickell, John Henry Burke, Melvil S. Coxe, Valentine Alphonsis Decot, Edward James Dourney, Harry Henry Ebberts, Harry Richard Silas Emes, John Fitz Gerald Fairbairn, Harry Nicholas Feltes, Thomas Francis Foley, Chauncey Wayne Grove, William Bentley Hamilton, B.B.E. and M.E.; Haley Waldo Hammond, Claude Smith Johnson, Lesser Kauffman, Ph.B.; Earl Dean Kilmer, Edward Charles Koenig, Arthur Garfield Lane, John C. S. Lappeus, Robert James Lawler, George Emerson Learn, Harry Roy Lohnes, Horace LoGrasso, William S. Lynch, Minor McDaniels, Myrtle Lothrop Massey, A.B.; William Michael Mehl, Robert C. Mehnert, Samuel Aloysius Moore, John Gray Morris, Jesse Clark Mosshammer, Stephen Vincent Mountain, John D'Arcy O'Brien, Caroline M. Osborn, John Corliss Plain, John Alphonse Ragone, Leonard Reu, Edna Augusta Rhodes, Victor Moreau Rice, Charles Richards, Floyd Richardson, Julius Richter, Herman William Schlappi, Charles Willard Selover, Robert Francis Sheehan, Jr., Douglass Henry Smith, Herbert Northrup Squier, Robert Shaw Taylor, Jr., Raymond Alexander Turnbull, John Lewis Van DeMark, George Garfield Wagner, Glenn Levi Whiting.

The honor roll was then announced by Dr. Mann, which included the following names, the standing of each being given; Chauncey Wayne Grove, Erie Pa., 92.51; Charles Richards, Buffalo, 92.54; Harry Roy Lohnes, Warren, Pa., 90.96; Earl Dean Kilmer, Rushford, 90.03.

DEPARTMENT OF PHARMACY.

The candidates for the degree of bachelor in pharmacy, thirty-eight in number, were then presented by Dr. John R. Gray, secretary of the college of pharmacy, and the chancellor conferred diplomas upon the following-named candidates:

William G. Beck, Henry A. Bell, Alfred J. Benson, Earl J. Brink, Cora May Brown, Walter L. Carlson, Ernest B. Cooper, Glen M. Coston, Homer E. Dyke, Charles J. Englehardt, Carl E. Freeman, Anna F. Frey, Oscar E. Heegaard, Walter K. Horton, George D. Hull, Sidney C. James, Lillian V. Jefferson, Louis A. Kaiser, Floyd M. Kline, Roland T. Lakey, Edith M. Lemon, Julian I. Le Roy, Edward S. Loge, Frank M. MacMurray, William H. McCoach, Otto S. McKee, Robert C. Mott, Anna C. W. Richter, Louis C. Sciarrino, J. Lee Sherlock, Glessner A. Smiley, John P. Smith, Albert D. Steadwell, Frank I. Strozzi, Clinton C. Turner, Willard E. Vincent, Clarence F. Walters, Eva H. Webster.

Dr. Willis G. Gregory, dean, then announced the honors and prizes in pharmacy as follows:

Senior honor roll—Mrs. Anna C. W. Richter, Buffalo, 96.90; Julian I. Le Roy, Poughkeepsie, 93.50; Homer E. Dyke, Port Angeles, Wash., 92.40; Miss Anna F. Frey, Warren, Pa., 90.10; Otto S. McKee, Lockport, 88.90.

Junior honor roll—Edgar H. Lincoln, Angelica; Arthur G. Drake, Cambridge Springs, Pa.; Wilber R. Davis, Norwich; W. A. Robinson, Buffalo; Rudolph W. Janke, Tonawanda.

Mrs. Anna C. W. Richter was awarded the Peabody prize of $50, and Edgar H. Lincoln that of the faculty junior prize of $25.

DEPARTMENT OF DENTISTRY.

The candidates for the degree of doctor in dental surgery, sixty-four in number, then were presented to the chancellor by Dr. George B. Snow, acting dean, and the following-named were given diplomas:

Clarence Robert Averill, Wesley Mason Backus, Lynn Berkeley Badgero, Ernest Rock Bailey, James Edward Barlow, Albert Wilson Beatty, Theron Coit Bliss, Glenn Kingston Brooks, Andrew Timothy Cahill, John Francis Clifford, Henry Graley Cody, Edgar Charles Cooke, Ward Hallock Cowles, Claude Francis Demerath, David Alanson Diltz, George Thomas Ellis, Carl Ferdinand Eschelman, Charles Augustus Espie, Alphaeus Vernon Fluhrer, John Frederick Folley, Elton Albert Foote, Leroy Herman Foote, Arthur James Foster, Walter Lowell Foster, Howard Thomas Gallagher, Donald Irving Gleason, Ernest Godfrey Graf, Warren Cogswell Greenfield, Mark A. Gregg, Fred J. Hall, Floyd Clinton Hart, Frank Morgan Howe, Daniel Edward Hurley, Howard Blish King, Francis Joseph Kelly, David Allen Lawton, Lloyd Elmer Leland, Frank Murel Leslie, James John Maloney, John Oppie McCall, A.B.; Daniel Francis McGillicuddy, Charles Michael McNeely, John Coatsworth Mesmer, Walter Lull Moore, Frank James Moyer, Jr., Garnet Howard Morden, Grover Cleveland Hayward Murdock, Alice Loretto Murphy, Robert Edmond Lee Northrup, Nathan Lee Otis, Earl Surrey Packwood, James Hadley Rasey, Herbert Elgene Read, Arthur Lawrence Reynolds, Clayton Alexander Sayers, Walter Evans Steacy, Frank House Tatlock, Clarence Albert Thorn, Peter Benjamin Van Wie, John Bernard West, Fred Bushnell Wilson, George Anson Wilson, John Ellsworth White, John Augustine Collins.

Honors were awarded as follows:

Magna cum laude—John Oppie McCall, Binghamton, 95.55.

Cum laude—Leroy H. Foote, Whitehall, 94.90; Lynn B. Badgero, Dayton, 94.81; Garnet H. Morden, Wellington, Ont., 94.55; Clayton Alexander Sayers, Watertown, 94.45; Grover C. H. Murdock, Simcoe, Ont., 94.27; Howard T. Gallagher, Schenectady, 93.90; John B. West, Keeneyville, Pa., 93.90; Floyd Clinton Hart, Sinclairville, 93.62; Donald I. Gleason, 93.18.

During the musical selection which followed, a feature of great interest to the audience and graduates consisted in the distribution of flowers. The ushers were kept busy for at least fifteen minutes in the performance of this duty, and many of the recipients of this delicate and beautiful attention were literally overladen with floral gifts. Cut flowers, garlands, and set pieces in profusion almost buried the graduates as they held their trophies during the remainder of the ceremonies.

The address to the graduated classes, delivered by Frank Hyatt Smith, M. A., of Buffalo, published elsewhere, was a masterful mingling of wisdom, wit and logic, interspersed with classic phrases and apt metaphors, which served to hold an impatient audience in fixed attention for more than half an hour. A benediction, followed by music, brought to a close these interesting and impressive commencement ceremonies of old Buffalo University.

